# Association between serum biomarkers and clinical stage in treatment-naïve chronic lymphocytic leukemia

**DOI:** 10.1016/j.htct.2026.106509

**Published:** 2026-08-06

**Authors:** Mohammadreza Khosravifarsani, Sobhan Parvizi, Hana Mohseni, Mohammad Tarbiat, Behnaz Zolfaghari, Mehrab Mostafaie, Sayyideh Forough Hosseini, Mohammad Ali Shafiee

**Affiliations:** a Department of Oncology, Cancer Prevention Research Center, Isfahan University of Medical Sciences, Isfahan, Iran; b Division of General Internal Medicine, Department of Medicine, University Health Network, Toronto General Hospital, Canada

**Keywords:** Chronic lymphocytic leukemia, Inflammatory markers, Rai stage, Prognosis

## Abstract

**Introduction:**

Chronic lymphocytic leukemia shows significant clinical heterogeneity, highlighting the need for reliable, accessible biomarkers for staging and prognosis, especially in resource-limited settings. This study evaluates the association between inflammatory and nutritional biomarkers and clinical stage in newly diagnosed, treatment-naïve patients.

**Methods:**

A cross-sectional study enrolled 150 treatment-naïve chronic lymphocytic leukemia patients and 150 matched healthy controls at outpatient hematology clinics affiliated with Isfahan University of Medical Sciences (2024–2025). Diagnoses followed International Workshop on chronic lymphocytic leukemia 2018 criteria. Controls were confirmed healthy by peripheral blood counts, smear evaluation, and clinical examination. The Rai staging system was used. Serum biomarkers were measured at diagnosis. Statistical analysis included Analysis of Variance and Kruskal-Wallis tests, and Spearman correlation.

**Results:**

Elevated ferritin, β2-microglobulin, lactate dehydrogenase and C-reactive protein levels, erythrocyte sedimentation rate, and C-reactive protein-to-albumin ratio correlated significantly with advanced Rai stages (III–IV) (p-value <0.05) reflecting greater tumor burden and systemic inflammation. Conversely, albumin and vitamin D3 levels declined with disease severity. Ferritin and β2-microglobulin showed the strongest correlations with clinical stages, reinforcing their prognostic value.

**Conclusion:**

Routine biomarkers such as ferritin, β2-microglobulin, lactate dehydrogenase, C-reactive protein-to-albumin ratio, and vitamin D3 are strongly associated with clinical stage and disease severity in treatment-naïve chronic lymphocytic leukemia patients. Their measurement provides cost-effective, accessible prognostic tools, especially where advanced molecular testing is unavailable. Incorporating these biomarkers into clinical staging may improve early risk stratification and guide management.

## Introduction

Chronic lymphocytic leukemia (CLL) is the most common form of adult leukemia in Western populations. It is characterized by the clonal proliferation and accumulation of small, mature-appearing B lymphocytes in the peripheral blood, bone marrow, and lymphoid tissues [1]. The clinical course of CLL is markedly heterogeneous, with some patients exhibiting indolent disease requiring no immediate therapy, while others experiencing rapid progression and resistance to conventional treatment modalities [2].

The CLL International Prognostic Index (CLL-IPI), incorporating five parameters (patient age, clinical stage, immunoglobulin heavy chain variable region (IGHV) mutation status, *TP53* gene status, and serum β2-microglobulin (β2-MG) levels) was introduced to address this clinical variability [3]. Of these, β2-MG, a low-molecular-weight protein associated with tumor burden and immune activation, has been widely validated as a prognostic biomarker in CLL and other lymphoid malignancies [4]. Similarly, *TP53* mutations and deletions are strongly correlated with poor treatment outcomes and reduced survival [5].

Beyond molecular determinants, systemic inflammation is increasingly recognized as a critical contributor to tumor biology in hematologic malignancies. Biomarkers such as C-reactive protein (CRP), albumin, ferritin, and composite indices like the CRP-to-albumin ratio (CAR) have demonstrated prognostic significance in various cancers, including CLL [3,6,7]. CAR, which reflects both systemic inflammation and nutritional status, has emerged as an independent predictor of overall survival in newly diagnosed CLL patients [3]. Albumin, a negative acute-phase reactant, typically declines in advanced disease and serves as a surrogate for nutritional depletion and chronic inflammation [3,6].

Ferritin, another acute-phase reactant, is elevated in a range of malignancies such as acute leukemias, multiple myeloma, and lymphomas [6., 7., 8.]. Ferritin not only reflects iron metabolism but also modulates immune responses, potentially contributing to immune evasion and leukemogenesis [7]. High serum ferritin levels have been shown to correlate with tumor mass and disease activity [6]. Vitamin D, a steroid hormone with immunomodulatory properties, has also been implicated in CLL pathogenesis. Deficiency of 25-hydroxyvitamin D3 [25(OH)D] is common among CLL patients and may predispose to immune dysfunction and increased infection risk [1].

While individual markers such as β2-MG, ferritin, CRP, and vitamin D have demonstrated prognostic relevance, limited research has explored their combined utility in treatment-naïve CLL patients. Investigating the interaction between molecular abnormalities and inflammatory or nutritional biomarkers could provide a more holistic and accessible approach to risk stratification, particularly in resource-constrained settings.

The present study aims to evaluate the association between selected inflammatory and nutritional biomarkers, including CRP, albumin, CAR, ferritin, vitamin D3 with clinical stage in newly diagnosed, untreated CLL patients. By integrating conventional clinical staging with biomarker profiles, this study seeks to elucidate their prognostic value and potential role in guiding early risk stratification.

## Materials and methods

### *Study design and population*

This cross-sectional observational study was conducted from January 2024 to December 2025 at outpatient hematology clinics affiliated with Isfahan University of Medical Sciences. A total of 150 newly diagnosed, treatment-naïve CLL patients were enrolled consecutively, based on the International Workshop on Chronic Lymphocytic Leukemia (IWCLL) 2018 diagnostic criteria, which define CLL by persistent B-cell lymphocytosis (≥5 × 10⁹/L for at least three months) and a characteristic immunophenotype confirmed by flow cytometry [9].

All patients were untreated before enrollment, that is, they had not received any CLL-directed therapy, corticosteroids, immunotherapy, or blood transfusions (including red blood cells and platelets). In order to reduce confounding effects, individuals with active infections, autoimmune disorders, chronic inflammatory diseases, or concurrent malignancies were excluded.

A control group of 150 healthy individuals, matched by age and sex, was recruited for comparative analysis. Control subjects did not meet the diagnostic criteria for CLL, as confirmed by peripheral blood counts, blood smear evaluation, and physical examination, including the absence of lymphadenopathy or organomegaly.

### *Clinical staging systems*

Disease severity was assessed using the Rai staging system, which is routinely used in clinical CLL management. The Rai staging system, introduced in 1975, classifies CLL into five stages (0–IV) based on progressive hematologic and clinical findings [10].

• Stage 0: Lymphocytosis only
• Stage I: Lymphocytosis with lymphadenopathy
• Stage II: Lymphocytosis with hepatomegaly and/or splenomegaly
• Stage III: Lymphocytosis with anemia (hemoglobin [Hb] <11 g/dL)
• Stage IV: Lymphocytosis with thrombocytopenia (platelet count <100 × 10⁹/L)

These stages are grouped into three prognostic categories: low risk (Stage 0), intermediate risk (Stages I–II), and high risk (Stages III–IV). The Rai system was selected in this study due to its widespread clinical utility. This study aimed to evaluate whether routinely available laboratory biomarkers could complement traditional staging systems by offering a more detailed and continuous understanding of disease burden across the CLL spectrum.

### *Data collection*

Collected data included demographics (age, sex, body mass index [BMI], place of residence), hematologic indices (white blood count [WBC], lymphocyte count, Hb, platelets), inflammatory and nutritional markers (CRP, erythrocyte sedimentation rate [ESR], albumin, lactate dehydrogenase [LDH], ferritin, iron, and vitamin D3), and molecular/immunologic markers such as β2-MG and the direct antiglobulin test (DAT). Flow cytometry was performed using a full diagnostic immunophenotyping panel, including CD19, CD5, CD23, CD20 (dim), CD43, CD79b, and surface immunoglobulin light chain restriction (kappa/lambda). The diagnosis of CLL was confirmed based on the co-expression of CD5 and CD23 on B-cells, dim CD20 expression, and light chain restriction, in line with World Health Organization (WHO) and IWCLL criteria. Flow cytometry markers were not included in the statistical analysis and served solely for diagnostic confirmation.

All clinical and laboratory data, including serum biomarker levels, were collected at the time of initial diagnosis and prior to any therapeutic intervention. All samples were obtained during a defined 12-month period with laboratory analyses being performed within 24 h of sample collection to minimize pre-analytical variability.

### *Laboratory procedures*

Laboratory assessments included complete blood count, biochemical measurements of CRP, ferritin, LDH, albumin, iron, and β2-MG, ESR (by Westergren method), serum vitamin D3 (by enzyme-linked immunosorbent assay), and flow cytometry for confirming CLL diagnosis.

## Statistical analysis

Continuous variables were summarized as means ± standard deviation or median with interquartile range depending on the distribution. One-way Analysis of Variance (ANOVA) was utilized to compare the means of normally distributed continuous variables across Rai stages, while the Kruskal-Wallis test was used for non-normally distributed variables. When the assumption of homogeneity of variances was violated, Welch's ANOVA was employed as an alternative statistical test. Post-hoc comparisons were done using the Dunn test for non-normally distributed variables. Continuous variables were visualized across Rai stages using box plots to explore distribution patterns. Multivariable linear regression models were performed to assess the association between each biomarker and Rai clinical stage, adjusting for age and sex. Stage 0 was used as the reference category. Categorical variables were compared across Rai stages using the Chi-square test. Data were analyzed using R software (version 4.5.0), and a p-value <0.05 was considered statistically significant.

### Ethical considerations

The study was approved by the Ethics Committee of Isfahan University of Medical Sciences (approval code: IR.SKUMS.REC.1403.163). All participants provided their written informed consent before enrollment.

## Results

The study included 150 newly diagnosed, treatment-naïve CLL patients, of whom 62.7% were male. The mean age of the patients was 64.08 ± 7.41 years. These patients were compared with 150 age- and sex-matched healthy controls. On comparing the Rai clinical stages, there was no statistically significant difference in mean ages (Welch’s ANOVA: p-value = 0.676), indicating demographic homogeneity across the clinical spectrum of patients.

Table 1 shows that serum biomarkers and hematological indices display significant differences in CLL patients compared to healthy controls. The serum ferritin, β2-MG, CRP, LDH, CAR, and ESR were significantly elevated in the patient group (all p-value <0.001), reflecting increased systemic inflammation due to tumour burden. On the other hand, albumin and vitamin D3 levels were markedly lower in CLL patients (p-value <0.001), suggesting chronic nutritional deficiency and potential immunomodulatory dysfunction. Hematological profiles revealed typical features of CLL, including anemia, thrombocytopenia, and pronounced leukocytosis, all consistent with underlying disease pathophysiology.

**Table 1:** Comparison of inflammatory and nutritional biomarkers between treatment-naïve chronic lymphocytic leukemia patients and healthy controls.

**Biomarker**	**Control Group (Mean ± SD)**	**CLL Patients (Mean ± SD)**	**p-value**
Ferritin (ng/mL)	180.4 ± 88.4	632.2 ± 437.5	<0.001
β2-MG (mg/L)	2.29 ± 0.75	4.39 ± 2.31	<0.001
Vitamin D3 (ng/mL)	36.4 ± 8.7	19.8 ± 7.7	<0.001
LDH (U/L)	228.8 ± 44.7	313.6 ± 117.6	<0.001
Albumin (g/dL)	4.28 ± 0.34	3.73 ± 0.63	<0.001
CRP (mg/L)	1.12 ± 1.06	7.07 ± 7.38	<0.001
CAR (CRP/Albumin)	0.26 ± 0.11	2.21 ± 3.01	<0.001
ESR (mm/h)	20.2 ± 7.4	39.5 ± 18.7	<0.001
Hb (g/dL)	14.1 ± 1.3	11.5 ± 2.3	<0.001
Platelets (×10⁹/L)	263.6 ± 54.3	246.9 ± 72.6	0.041
WBC (×10⁹/L)	7.3 ± 2.0	24.4 ± 15.5	<0.001

β2-MG: serum β2-microglobulin; LDH: Lactate dehydrogenase; ESR: Erythrocyte sedimentation rate; CRP: C-reactive protein; CAR: C-reactive protein-to-albumin ratio; Hb: Hemoglobin; WBC: White blood cell count

Progressive changes in biomarker levels were observed across Rai stages, highlighting their potential utility in clinical disease stratification (Table 2).

**Table 2:** Comparison of serum biomarkers across Rai clinical stages in treatment-naïve chronic lymphocytic leukemia patients.

**Biomarker**	**Stage 0 (Mean ± SD)**	**Stage I (Mean ± SD)**	**Stage II (Mean ± SD)**	**Stage III (Mean ± SD)**	**Stage IV (Mean ± SD)**	**p-value**
Ferritin (ng/mL)	154.03 ± 52.60	376.17 ± 226.55	484.81 ± 199.36	1126.90 ± 500.35	889.60 ± 590.68	<0.001
β2-MG (mg/L)	2.53 ± 0.56	2.20 ± 0.40	3.67 ± 0.50	5.49 ± 0.53	8.05 ± 1.07	<0.001
LDH (U/L)	199.60 ± 25.50	203.65 ± 18.51	256.80 ± 24.07	457.17 ± 90.51	448.66 ± 108.73	<0.001
Albumin (g/dL)	4.21 ± 0.36	4.48 ± 0.33	3.94 ± 0.39	3.45 ± 0.29	2.66 ± 0.45	<0.001
ESR (mm/h)	21.94 ± 7.28	20.31 ± 5.96	39.32 ± 6.60	63.86 ± 8.99	94.07 ± 9.18	<0.001
CRP (mg/L)	2.61 ± 1.48	5.55 ± 2.67	3.10 ± 1.41	9.38 ± 2.88	32.86 ± 10.56	<0.001
CAR	0.62 ± 0.34	0.01 ± 0.01	0.05 ± 0.01	0.16 ± 0.07	1.23 ± 0.49	<0.001
Hb (g/dL)	14.50 ± 1.19	14.05 ± 1.34	12.83 ± 1.33	10.61 ± 0.88	8.08 ± 1.01	<0.001
Platelet count (×10⁹/L)	293 ± 53.88	290.51 ± 77.69	250.06 ± 69.16	111.76 ± 18.65	65.73 ± 19.13	<0.001
WBC count (×10⁹/L)	30.71 ± 9.75	30.48 ± 11.08	77.61 ± 14.66	144.96 ± 27.81	346.81 ± 34.52	<0.001
Vitamin D3 (ng/mL)	30.85 ± 6.26	21.58 ± 9.20	18.57 ± 8.56	14.43 ± 5.62	12.72 ± 4.17	<0.001

SD: Standard deviation; β2-MG: β2-Microglobulin; LDH: Lactate dehydrogenase; ESR: Erythrocyte sedimentation rate; CRP: C-reactive protein; CAR: C-reactive protein-to-albumin ratio; Hb: Hemoglobin; WBC: White blood cell count

In multivariable linear regression analyses, levels of ferritin, β2-MG, LDH, ESR, CRP, and the CRP/albumin ratio, as well as WBC and platelet count, were significantly associated with CLL stages after adjusting for age and sex. Ferritin, β2-MG, LDH, ESR and CRP levels, and WBC progressively increased with advancing stage, while albumin, Hb, vitamin D3, and platelet counts showed significant declines, particularly in stages III and IV (p-values <0.01 for all comparisons). Age and sex were not significant predictors in any of the models. These results underscore the independent prognostic value of these routinely accessible biomarkers in staging and monitoring CLL progression.

**Figure 1:**
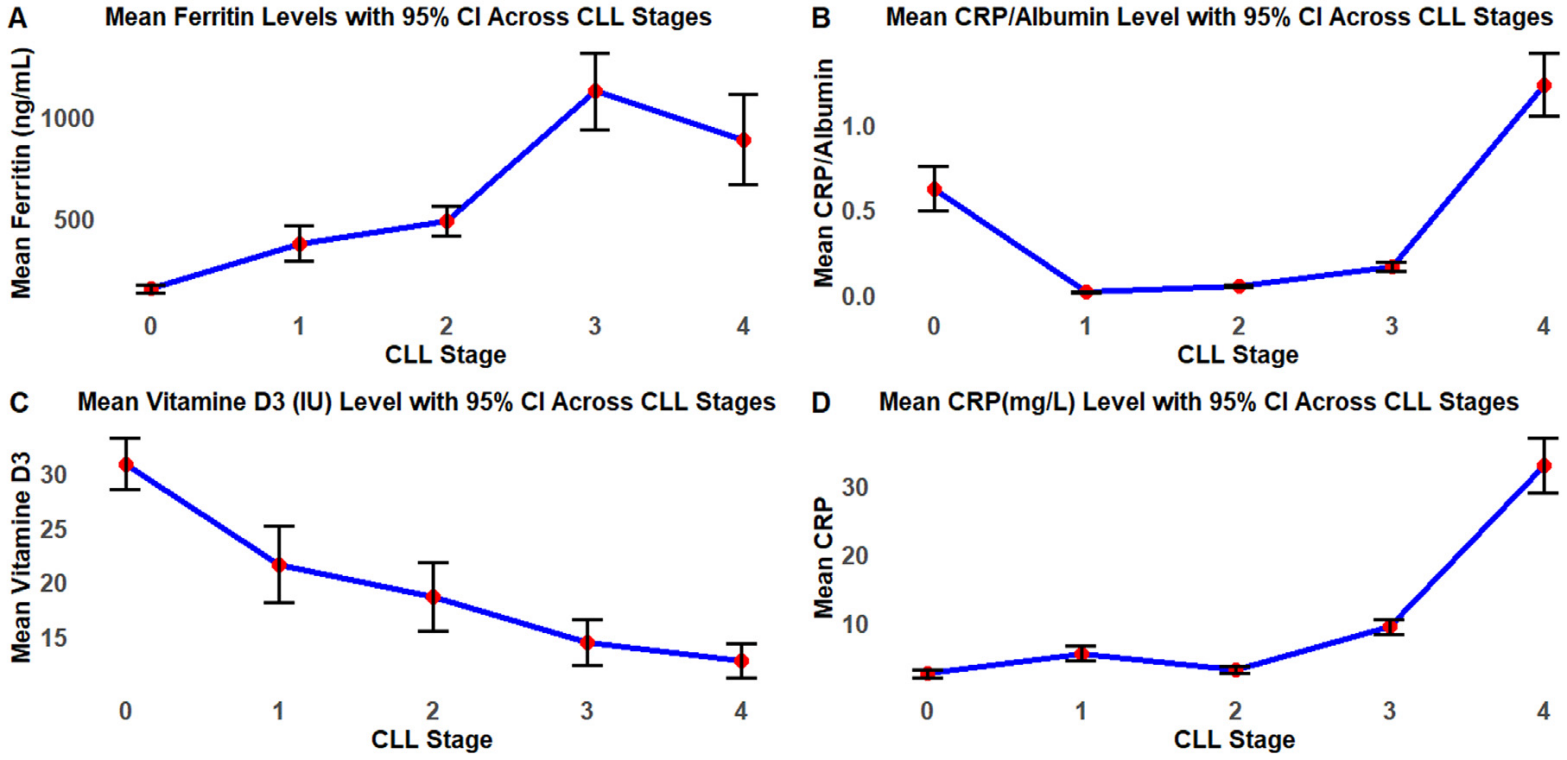
Serum biomarker trends across Rai stages in treatment-naïve chronic lymphocytic leukemia (CLL) patients. A) Mean Ferritin Levels (ng/mL) increase progressively with disease stage, peaking at Stage III and declining slightly at Stage IV, indicating its correlation with tumor burden. B) C-reactive protein–to-albumin ratio CAR demonstrates early-stage fluctuation followed by a sharp rise at Stage IV, underscoring systemic inflammation and nutritional compromise in advanced disease. C) Vitamin D3 Levels (ng/mL) decline steadily from Stage 0 to Stage IV, supporting its role in immunologic dysfunction and disease severity. D) C-reactive protein (CRP) levels (mg/L) show a progressive and significant increase across Rai stages, aligning with systemic inflammatory progression in CLL.

Ferritin (Figure 1), β2-MG, and LDH levels increased significantly from Rai Stage 0 through Stage III, with a slight decline or plateau observed at Stage IV (p-value <0.001). These patterns support their role as surrogate markers for tumour proliferation and metabolic activity.

The CAR (Figure 1), a composite index of inflammation and nutritional status, was markedly elevated in Stages III and IV (p-value <0.001). Hematologic parameters worsened with advancing stage, characterized by increased severity of anemia and thrombocytopenia, as well as higher WBC counts (p < 0.001), reflecting disease progression.

Albumin levels declined steadily with disease progression, decreasing from 4.21 g/dL in Stage 0 to 2.66 g/dL in Stage IV (p-value <0.001), indicative of worsening nutritional status and increased systemic inflammation. Similarly, vitamin D3 levels showed a continuous downward trend from 30.85 ng/mL in Stage 0 to 12.72 ng/mL in Stage IV (p-value <0.001), reinforcing its relevance as a prognostic marker of immune dysregulation in CLL (Figure 1)

Markers of inflammation, including CRP (Figure 1) and ESR, increased substantially in later Rai stages (p-value <0.001), consistent with an intensifying inflammatory response in advanced disease.

All values are presented as means with 95% confidence intervals

### *Patient demographics and baseline characteristics*

This study included 150 newly diagnosed, treatment-naïve CLL patients (62.7% male; mean age: 64.08 ± 7.41 years) alongside 150 age- and sex-matched healthy controls. No significant difference in mean age was observed across Rai stages (Welch’s ANOVA: p-value = 0.676), indicating demographic homogeneity across the clinical spectrum of disease.

### *Comparison with healthycontrols*

As shown in Table 1, serum levels of ferritin, β2-MG, CRP, LDH, CAR, and ESR were significantly elevated in CLL patients compared to healthy individuals (all p-value <0.001) reflecting increased tumor burden and systemic inflammation. In contrast, albumin and vitamin D3 levels were markedly reduced, suggesting chronic nutritional deficiency and immunomodulatory dysfunction. Hematological indices further revealed anemia, thrombocytopenia, and marked leukocytosis, consistent with CLL pathophysiology.

### *Stage-specific biomarker trends in chronic lymphocytic leukemia*

Progressive alterations in biomarker profiles were observed across Rai clinical stages (Table 2), underscoring the utility of serum-based indicators for disease stratification.

Ferritin, β2-MG, and LDH levels demonstrated a statistically significant upward trend across Rai stages, reaching their highest values in Stage IV (p-value <0.001). This trajectory supports their role as surrogate markers of tumor proliferation and metabolic activity (p-value <0.001). Albumin levels exhibited a steady decline with advancing stages, from 4.21 g/dL (Stage 0) to 2.66 g/dL (Stage 4), signifying deteriorating nutritional and inflammatory status (p-value <0.001). Similarly, vitamin D3 levels fell consistently across stages, from 30.85 ng/mL at Stage 0 to 12.72 ng/mL at Stage 4, reinforcing its prognostic relevance in immune dysregulation (p-value <0.001). CRP and ESR values rose significantly in later stages, highlighting the intensifying inflammatory milieu in advanced CLL (p-value <0.001). The CAR showed marked elevation in Stages III–IV (p-value <0.001), offering a composite index of both inflammation and nutritional decline. Hematologic trends showed worsening anemia, thrombocytopenia, and escalating WBC counts with higher Rai stages (p-value <0.001), further corroborating disease progression.

These findings confirm that a combination of inflammatory, nutritional, and hematologic biomarkers can effectively reflect disease severity and staging in CLL.

## Discussion

This study aimed to determine whether commonly available inflammatory and nutritional biomarkers, including ferritin, CRP, albumin, the CAR, LDH, ESR, and vitamin D3, are associated with clinical stage and disease burden in newly diagnosed, treatment-naïve CLL patients. The findings of this research indicate that elevated levels of ferritin, CRP, ESR, LDH, and CAR were significantly associated with more advanced Rai stages, while serum albumin and vitamin D3 levels showed a progressive decline with increasing disease severity. Among these, ferritin and CAR emerged as particularly strong surrogate markers of tumor burden and systemic inflammation, consistent with prior research highlighting their prognostic relevance in hematologic malignancies [3]. These results reinforce the potential utility of these routine laboratory biomarkers in the initial risk stratification of CLL, particularly in resource-constrained settings where molecular testing may not be readily available.

In the current cohort of newly diagnosed, treatment-naïve CLL patients, elevated serum ferritin levels were significantly associated with more advanced clinical stages. This finding suggests that ferritin may serve as a surrogate marker of both tumor burden and disease activity. The results are consistent with prior research in hematologic malignancies such as acute myeloid leukemia, non-Hodgkin lymphoma, and multiple myeloma, in which high ferritin levels have been linked to more aggressive disease and poorer survival outcomes [8,11,12]. The mechanistic basis for this association is multifactorial: ferritin reflects not only systemic inflammation and iron overload, but also plays a role in tumor biology through oxidative stress modulation, impairment of lymphocyte differentiation, and immune evasion [7,13]. However, the prognostic role of ferritin in CLL remains somewhat controversial, with some studies failing to show a consistent correlation between ferritin and disease severity [14]. This variability may reflect differences in patient selection, presence of comorbidities such as autoimmune hemolytic anemia or liver dysfunction, and timing of biomarker measurement relative to disease progression. Furthermore, given the role of ferritin as an acute-phase reactant, elevated levels may sometimes reflect non-malignant inflammatory processes, potentially limiting its specificity in early-stage or indolent CLL. Nonetheless, the consistent elevation of ferritin in advanced stages across hematologic cancers supports its integration alongside other prognostic markers such as β2-MG, CAR, and *TP53* mutation status in risk assessment models.

A growing body of evidence also points to the prognostic significance of vitamin D3 in CLL. In the present study, vitamin D3 levels declined progressively with increasing Rai stage, a finding that aligns with prior reports documenting widespread vitamin D deficiency among CLL patients. Shanafelt et al. were among the first to demonstrate that 25(OH)D levels below 25 ng/mL were associated with nearly twice the risk of disease progression and shorter time to first treatment [15]. Subsequent studies have validated these observations. For instance, Ito et al. found that low vitamin D3 levels correlated significantly with high β2-MG levels and advanced Rai/Binet stages [16]. The biological rationale is grounded in the immunoregulatory function of vitamin D: its active form, 1,25-dihydroxyvitamin D, modulates immune cell activity by enhancing cytotoxic T cells and natural killer cell function while suppressing pro-inflammatory cytokines [17]. These mechanisms are particularly relevant in CLL, which is characterized by chronic immune dysregulation and impaired immune surveillance. Despite compelling observational data, there is a lack of large-scale randomized controlled trials evaluating the impact of vitamin D supplementation on clinical outcomes in CLL. Whether deficiency is a causative factor or merely a marker of disease burden remains unclear, but vitamin D assessment may offer a valuable component of initial prognostic evaluation.

The findings of this study also confirmed that LDH levels were significantly elevated in patients with advanced CLL, supporting its role as a surrogate marker for tumor burden and cellular turnover, as highlighted in several previous studies [18., 19., 20.]. A stage-wise increase in LDH from Rai 0 to IV was observed in the present cohort, consistent with the previous pattern [18]. Although LDH is not included in the CLL-IPI, these results suggest that it has clinical relevance as a reflection of metabolic activity and tumor dynamics. Additionally, inflammatory markers such as ESR and CRP showed progressive elevations with advancing disease stage, corroborating findings from earlier studies by Conroy et al. and Hallek et al., who identified these markers as reflective of systemic inflammatory load in lymphoproliferative disorders [1,21]. The data here also showed increased prevalence of hypoalbuminemia in higher Rai stages, reinforcing the value of albumin as a negative prognostic factor, particularly when interpreted in the context of CRP. The CAR, a novel index incorporating both inflammation and nutritional status, was significantly elevated in patients with progressive disease, echoing previous findings by Tang et al. [3]. These integrated trends suggest that a composite panel involving LDH, CRP, ESR, albumin, and CAR could provide a more comprehensive and accessible risk stratification model. Discrepancies with earlier studies may reflect population heterogeneity, comorbidity profiles, or stage distribution. Future prospective studies are warranted to validate the clinical utility of these biomarkers in broader, real-world settings.

Although both Rai and Binet staging systems are used interchangeably in CLL prognostication, the findings of this study showed a more consistent and progressive association between biomarker levels and Rai stage. This may be due to the greater staging resolution offered by the five-tier Rai system, which enables more precise stratification of laboratory and inflammatory markers, particularly in early-stage disease. In contrast, the broader grouping in the Binet system may obscure intermediate biological variations. These findings suggest that, for biomarker-based prognostic modeling, Rai staging may provide more granularity for research purposes, especially in early or indolent CLL.

Beyond classical tumor burden and inflammatory biomarkers, growing attention has been directed toward evaluating the role of BMI and geographic residence in the pathogenesis and progression of CLL. Several large, population-based cohort studies have identified a modest but significant association between elevated BMI and increased risk of developing CLL, particularly among males [22,23]. A meta-analysis further quantified this risk, revealing a 25% increase in CLL incidence for every 5 kg/m² rise in BMI [24]. The underlying mechanisms are hypothesized to involve obesity-induced immune dysregulation, chronic low-grade inflammation, and dysregulated cytokine signaling, all of which may contribute to lymphomagenesis [25,26].

Despite these associations, BMI has not been incorporated into major prognostic models such as the CLL-IPI, and most clinical investigations have failed to establish a robust link between BMI and disease stage, cytogenetic abnormalities, or treatment response [1,27]. While not a direct surrogate for disease severity, BMI may impact broader clinical variables including treatment tolerance, immune competence, and metabolic health, especially in older or comorbid populations [28].

The potential influence of place of residence, specifically urban versus rural environments, has also been explored in epidemiologic studies, though findings remain largely indirect. Geographic variation in CLL incidence has been observed in registry-based datasets, which may reflect differences in environmental exposures, healthcare access, and diagnostic intensity, rather than intrinsic biological differences [22,23]. Rural residence has been associated with increased exposure to pesticides, zoonotic agents, and radon, each of which may elevate CLL risk [24,26,27]. Moreover, individuals living in rural settings may face barriers to early diagnosis, molecular testing, and specialist access, which could influence disease progression and care quality [1,25,29]. Nonetheless, current evidence does not demonstrate a consistent correlation between residence type and CLL stage at diagnosis. These insights suggest that while BMI and geographic factors may not directly affect clinical staging, they may influence disease risk, access to care, and long-term outcomes, thereby warranting further investigation through large-scale, prospective studies.

Although patient characteristics such as BMI and residence (urban vs. rural) were recorded, analysis did not identify significant correlations between these factors and clinical stage. However, future research in larger cohorts may better elucidate the role of such demographic factors in disease progression.

While this study did not assess regional prevalence patterns of CLL, we acknowledge that this represents an important gap in the literature, particularly in underrepresented non-Western populations. Future investigations should aim to clarify how geographic variation may shape disease burden and biomarker expression.

### *Strengths and limitations*

This study has several notable strengths. It offers a comprehensive evaluation of multiple accessible serum biomarkers, including ferritin, CRP, albumin, LDH, ESR, vitamin D3, and the CAR, within a well-defined cohort of newly diagnosed, treatment-naïve CLL patients. The selection of this population eliminates potential confounding from prior therapies and provides clean baseline data reflective of the untreated disease state. Additionally, the use of standardized clinical staging via the Rai system enables meaningful correlation with disease burden and improves comparability with other studies. Importantly, the biomarkers evaluated are cost-effective, widely available, and routinely measured, enhancing the clinical applicability of the findings, particularly in resource-limited settings. Furthermore, their incorporation into routine clinical practice may facilitate earlier risk stratification in regions where advanced molecular testing is not readily accessible.

However, several limitations must be acknowledged. The cross-sectional design restricts the ability to assess temporal trends, causality, or changes in biomarker levels over time. The modest sample size may also limit statistical power and reduce generalizability to larger or more diverse populations. Another limitation is the absence of molecular and cytogenetic profiling (e.g., *IGHV* mutation status, *TP53* disruption), which are key components of modern CLL risk stratification. In addition, the specificity of inflammatory markers such as CRP, ESR, and ferritin could be affected by subclinical infections, autoimmune conditions, or other comorbid inflammatory states. Lastly, the study lacks longitudinal outcome data, including progression-free or overall survival, which prevents confirmation of the prognostic utility of these biomarkers. Future prospective, longitudinal studies incorporating molecular risk factors and survival endpoints are warranted to validate and expand upon these preliminary findings.

## Conclusion

This study highlights the clinical relevance of readily available inflammatory and nutritional biomarkers, such as ferritin, β2-MG, LDH, CRP, albumin, CAR, ESR, and vitamin D3, in relation to disease burden and staging in treatment-naïve CLL patients. The findings of the present study demonstrate that elevated levels of ferritin, β2-MG, LDH, CRP, ESR, and CAR are significantly associated with advanced Rai stages, while declining albumin and vitamin D3 levels reflect worsening nutritional and immunologic status.

Among these, ferritin and β2-MG emerged as particularly strong indicators of tumor burden, while CAR and vitamin D3 levels may reflect the broader interplay between systemic inflammation and immune dysregulation. These routinely measured, cost-effective biomarkers have the potential to enhance initial risk stratification and clinical decision-making, especially in settings where molecular diagnostics are limited. Their integration into clinical assessment frameworks may enhance early risk stratification and management in resource-constrained environments.

Future prospective, longitudinal studies incorporating survival outcomes and molecular profiles are warranted to validate the prognostic value of these biomarkers and assess their integration into existing clinical models for individualized patient management in CLL.

## Conflicts of interest

The authors declare that they have no known competing financial interests or personal relationships that could have appeared to influence the work reported in this paper.
